# Cardiovascular Disease May Be Triggered by Gut Microbiota, Microbial Metabolites, Gut Wall Reactions, and Inflammation

**DOI:** 10.3390/ijms251910634

**Published:** 2024-10-02

**Authors:** Leon M. T. Dicks

**Affiliations:** Department of Microbiology, Stellenbosch University, Stellenbosch 7600, South Africa; lmtd@sun.ac.za

**Keywords:** cardiovascular disease, gut microbiota, gut wall reactions, inflammation

## Abstract

Cardiovascular disease (CVD) may be inherited, as recently shown with the identification of single nucleotide polymorphisms (SNPs or “snips”) on a 250 kb DNA fragment that encodes 92 proteins associated with CVD. CVD is also triggered by microbial dysbiosis, microbial metabolites, metabolic disorders, and inflammatory intestinal epithelial cells (IECs). The epithelial cellular adhesion molecule (Ep-CAM) and trefoil factor 3 (TFF3) peptide keeps the gut wall intact and healthy. Variations in Ep-CAM levels are directly linked to changes in the gut microbiome. Leptin, plasminogen activator inhibitor 1 (PAI1), and alpha-1 acid glycoprotein 1 (AGP1) are associated with obesity and may be used as biomarkers. Although contactin 1 (CNTN1) is also associated with obesity and adiposity, it regulates the bacterial metabolism of tryptophan (Trp) and thus appetite. A decrease in CNTN1 may serve as an early warning of CVD. Short-chain fatty acids (SCFAs) produced by gut microbiota inhibit pro-inflammatory cytokines and damage vascular integrity. Trimethylamine N-oxide (TMAO), produced by gut microbiota, activates inflammatory Nod-like receptors (NLRs) such as Nod-like receptor protein 3 (NLRP3), which increase platelet formation. Mutations in the elastin gene (*ELN*) cause supra valvular aortic stenosis (SVAS), defined as the thickening of the arterial wall. Many of the genes expressed by human cells are regulated by gut microbiota. The identification of new molecular markers is crucial for the prevention of CVD and the development of new therapeutic strategies. This review summarizes the causes of CVD and identifies possible CVD markers.

## 1. Introduction

Cardiovascular disease (CVD) is mostly associated with an unhealthy diet that leads to obesity, coronary sickness, stroke, and diabetes, but may also be genetically inherited. However, recent studies have shown that CVD is a complex multifactorial disease that involves metabolic disorders and inflammatory reactions of various organs, including the liver, intestine, and central nervous system (CNS). CVD should thus be defined as a ‘systemic disease’ rather than a single-organ disease. Genomic and metagenomic studies group CVD in the same category as other systemic diseases such as type 2 diabetes [1], inflammatory bowel disease (IBD) [2,3], and certain cancers [4]. Most studies were conducted in isolation, focusing on a specific organ or a group of gut microbiota. Few studies addressed the molecular changes in plasma that develop during interactions between gut microbiota, organs, and the human genome.

The vast majority of human microbiota (97%) is found in the gastrointestinal tract (GIT), largely in the colon [5,6]. More than 90% of the human gut microbiome, close to 2800 species, belong to the phyla Proteobacteria, Firmicutes, Actinobacteria, and Bacteroidetes [7,8,9,10,11,12,13]. The remaining 10% of the gut microbiome comprises Fusobacteria and Verrucomicrobia [14]. Despite numerous studies, 60–80% of gut bacteria have not been cultured. Their phenotypic properties remain unknown, including their role in the GIT, gut homeostasis, regulation of the immune system, maintenance of intestinal epithelial cells (IECs), regulation of neurological and endocrine functions, and CVD [15,16]. Neurotransmitters such as γ-aminobutyric acid (GABA), norepinephrine (NE), serotonin (5-HT), dopamine (DA), short-chain fatty acids (SCFAs) [17], glutamate (Glu), tryptophan (Trp) [18], histamine (His) [19], and secondary bile acids [20] produced by gut bacteria reach the CNS. In turn, signals from the brain communicate with enterochromaffin cells (ECs) and enteroendocrine cells (EECs) in the gut wall and the mucosal immune system [21]. Communication between the gut and the brain (gut–brain axis, GBA) improves the integrity of the gut wall, reduces peripheral inflammation, and inhibits the release of pro-inflammatory cytokines [22]. Signals generated by the hypothalamus, in response to metabolites produced by gut microbiota, reach the pituitary and adrenal glands and communicate with EECs via the hypothalamic–pituitary–adrenal axis (HPA) [23]. The role gut microbiota play in CVD is not well understood.

**Figure 1:**
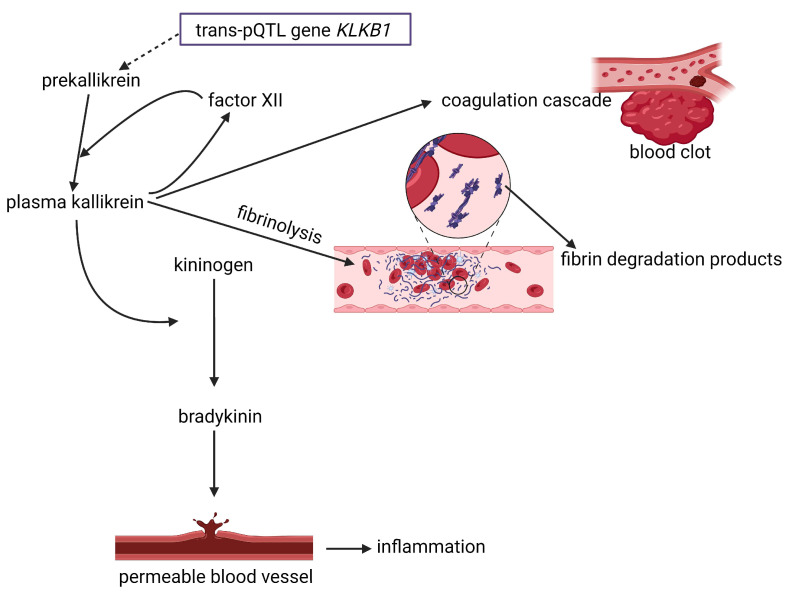
A basic explanation for the role of plasma kallikrein in inflammation and blot clotting. Prekallikrein is encoded by the trans-pQTL gene *KLKB1* in the liver. Factor XII converts prekallikrein to plasma kallikrein and leads to the production of more factor XI. The early stages of blood clotting and the formation of bradykinin are initiated by a combined reaction of plasma kallikrein and factor XII. Bradykinin increases the permeability of blood vessel walls, leading to leakage and inflammation. Trans-pQTL = trans-protein quantitative trait loci. Created using Biorender.com (accessed on 22 August 2024).

A genetic study by Zhernakova et al. [24] shed some light on the association between gut microbiota and CVD. The authors performed a protein quantitative trait loci (pQTLs) analysis by testing the single nucleotide polymorphisms (SNPs or “snips”) located within a 250 kb DNA fragment encoding 92 CVD-related proteins. Mapping pQTLs far from the cognate gene (a gene coding for an essential protein constantly produced) or on a different chromosome (trans-pQTLs) yielded 85 disease-associated proteins. Of these, 19 cis-pQTLs (SNPs located within 1 Mb upstream or downstream of the corresponding protein-coding gene’s transcription start site), and 74 trans-pQTLs were associated with complex traits and diseases. At least 10 cis- and 7 trans-regulated proteins were associated with CVD. The analyses of 422 putative CVD-associated SNPs revealed 14 pQTLs, each encoding a protein that may be involved in regulating CVD genes [24]. An example is the trans-pQTL gene *KLKB1*, which codes for prekallikrein (a serine protease) in the liver [25] (Figure 1). Factor XII, a protease that is mainly produced in the liver, converts prekallikrein to plasma kallikrein (a subgroup of serine proteases found in human urine) that has hypotensive properties and produces more factor XII [26] (Figure 1). Plasma kallikrein and factor XII initiate the early stages of coagulation and blood clotting [26]. The interaction between plasma kallikrein and factor XII also initiates a series of chemical reactions that lead to the release of the nanopeptide bradykinin [27] (Figure 1). The latter increases the permeability of blood vessel walls, leading to leakage and inflammation [26]. Several plasma proteins are linked to the gut microbiome, e.g., plasminogen activator inhibitor (PAI)-1, that decreases fibrinolysis and promotes clot formation; urokinase plasminogen activator (uPA), a serine protease that converts inactive plasminogen into active plasmin; epidermal growth factor receptor (EGFR), a protein involved in cell signaling pathways, cell division, and survival; paraoxonases (PONs), especially PON1, which protects LDL and outer cell membranes against harmful oxidative modifications; tumor necrosis factor receptor 2 (TNF-R2), which has pro- and anti-inflammatory properties protecting oligodendrocytes, cardiomyocytes, and keratinocytes; and insulin-like growth factor (IGF), a hormone that controls the production of growth hormones (GHs) and promotes the growth of bones and tissues [24]. Although an individual’s genetic makeup contributes most strongly to immune-stimulating proteins, the gut microbiome takes the lead in the production of proteins involved in metabolism and obesity-related inflammation [24]. Based on these findings, intestinal health, lipid oxidation, and the CNS play a critical role in CVD. Much more research is required to fully understand the interactions between host (human) genetics and the gut microbiome.

This review focuses on the different factors involved in CVD. The review does not intend to cover all of the aspects in depth, but instead provide the reader with a broad overview of how inflammation may lead to CVD, the influence of eating disorders, and the role gut microbiota and short-chain fatty acids play in inflammation. The possible markers that may be used to detect CVD are pointed out.

## 2. The Role of Epithelial Cellular Adhesion Molecule (Ep-CAM) and Trefoil Factor 3 (TFF3) in Inflammation and Cardiovascular Disease (CVD)

Two proteins attracting much attention in CVD research are Ep-CAM (epithelial cellular adhesion molecule) and the small peptide trefoil factor 3 (TFF3) secreted by intestinal goblet cells. The function of Ep-CAM is to keep intestinal epithelial cells (IECs) in close contact by acting as a “sticking agent” that also prevents mucosal infections. Ep-CAM-deficient mice developed increased intestinal permeability accompanied by a decrease in ion transport, both associated with CVD [28]. The study of Zhernakova et al. [24] provided evidence for an interplay between genetics, the gut microbiome, and Ep-CAM plasma levels. As much as 26.5% of the variation in Ep-CAM levels is associated with changes in gut microbiota patterns and metabolic processes [24]. Only 7% of the changes in Ep-CAM levels are linked to two trans-pQTL loci [24]. The trans-pQTL locus *FUT2* is also associated with changes in plasma vitamin B12 levels, microbial infections, and Crohn’s disease (CD). Changes in Ep-CAM levels affect the impact of FUT2 on butyrate-producing *Blautia* spp. [24], which has a significant correlation with obesity, diabetes, cancer, and various inflammatory diseases [29]. TFF3 plays a role in repairing the intestinal mucosa [30]. However, more recent research has linked TFF3 to cancer, colitis, gastric ulcers, diabetes mellitus, non-alcoholic fatty liver disease (NAFLD), and abnormalities of the CNS [30]. Despite these observations, the biological functions and specific modes of action of TFF3 in the respective tissues have not been elucidated (for further information, the reader is referred to Yang et al. [30]). Further research on the molecular function of TFF3 in specific diseases may lead to the development of novel therapeutic drugs.

## 3. Obesity, Proteins Contactin 1 (CNTN1), Notch 3, and Elastin

Obesity, which can be defined as the accumulation of lipids, is often associated with elderly people, insulin resistance, hyperlipidemia, hypertension, metabolic syndrome (MetS), and chronic low-grade inflammations [31]. Of these symptoms, obesity, and MetS are directly linked to increased CVD [32]. Other factors contributing to the risk of developing CVD are excessive alcohol consumption, smoking, exposure to pollutants and carcinogens, inactivity, depression, persistent anxiety, and hereditary and ethnic background [33]. Visceral adipose tissue (VAT), subcutaneous adipose tissue (SAT), pericardial fat, and intrathoracic fat are all associated with metabolic risk, with VAT being the strongest associated with cardiometabolic risk and CVD [32]. The authors identified four biomarkers (leptin, plasminogen activator inhibitor 1 (PAI1), alpha-1 acid glycoprotein 1 (AGP1), and CNTN1) for obesity and adiposity. All except CNTN1 were positively associated with obesity and adiposity [32]. The role of AGP1 and CNTN1 in cardiometabolic disease is ill-defined [32]. The CNTN1 gene (*CNTN1*) encodes an unknown regulator with a key function in the CNS. There is a clear association between CNTN1 and the bacterial metabolism of Trp [24]. Trp is important in appetite regulation and is linked to the CNS via the GBA [34,35]. The decrease in plasma concentrations of CNTN1 in obese individuals may be used as a marker for the early detection of CVD [36].

Diseases such as hypertension, atherosclerosis, vascular stenosis, and dilation/thinning of the vascular wall that causes aneurysms are characterized by changes in the proliferation and differentiation of vascular smooth muscle cells (VSMCs) [37]. Notch 3 protein, encoded by the gene *NOTCH3*, plays a pivotal role in maintaining VSMCs surrounding blood vessels and gene transcription regulation [37]. The one domain of Notch 3 remains inside the cell, the middle domain spans across the cell membrane, and the extracellular domain protrudes from the cell to attach to other proteins (ligands) [38]. Upon binding to ligands, the intracellular domain detaches from the rest of the protein, now defined as the Notch intracellular domain (NICD), enters the cell nucleus, and regulates gene transcriptions [38]. NOTCH3 plays an important role in intracellular communication, neural development, and binding of epidermal growth factor (EGF) to its receptor, EGFR. EGF and tumor growth factor α (TGFα) stimulate the division of multiple cell types in the gastrointestinal tract and enhance mucosal healing and angiogenesis [39].

Elastin, one of the most abundant proteins in the human body and a major component of the extracellular matrix, constitutes a large part of blood vessel walls. Mutations in the elastin gene (*ELN*) cause supra valvular aortic stenosis (SVAS), defined as the thickening of the arterial wall. This leads to increased stenosis of arteries, including coronary arteries [40]. Although deficiencies or the degradation of elastin do not cause atherosclerosis, it has an indirect effect by allowing for the formation of lipid plaques in arteries [41].

## 4. Lipoproteins and Paraoxonases (PONS)

**Figure 2:**
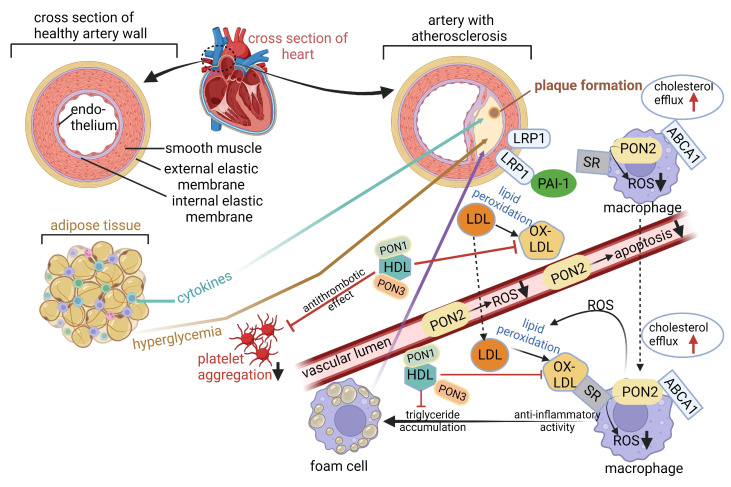
A schematic representation explaining the role paraoxonases (PON1, PON2, and PON3) play in cardiovascular disease (CVD). PON1 and PON3 are attached to high-density lipoprotein (HDL) and oxidize lipid peroxides to prevent their accumulation on low-density lipoprotein (LDL). Oxidized LDL (OX-LDL) triggers macrophages to an anti-inflammatory state and forms foam cells. Foam cells and the release of pro-inflammatory cytokines from adipose tissue lead to plaque formation. Plague formation may also be triggered by hyperglycemia. The HDL-PON complex prevents the aggregation of platelets. PON2 represses the formation of reactive oxygen species (ROS) and has an antiatherogenic effect. ATP-binding cassette transporter A1 (ABCA1) mediates the cellular efflux of phospholipids and cholesterol to lipid-poor apolipoprotein A1 (apoA1)-HDL and plays a significant role in the metabolism of HDL. Blue dots in adipose tissue represent cytokines, lighter circles in the foam cell represent the accumulation of triglycerides, the yellow area in the artery represents hyperglycemia and atherosclerosis, LRP1 = low-density lipoprotein receptor-related protein 1, PAI1 = plasminogen activator inhibitor 1, ABCA1 = ATP-binding cassette transporter A1, SR = scavenger receptor. Created using Biorender.com (accessed on 20 August 2024).

The oxidation of low-density lipoprotein (LDL or “bad” cholesterol) increases the risk of developing CVD (https://www.cdc.gov/cholesterol/about/ldl-and-hdl-cholesterol-and-triglycerides.html; accessed on 15 August 2024). Oxidation is executed by paraoxonases (PONS) attached to high-density lipoprotein (HDL or “good” cholesterol) [42], as displayed in Figure 2. PON1 and PON3 are associated with HDL. PON1 oxidizes lipid peroxides to prevent their accumulation on LDL (Figure 2). PON1 regulates reverse cholesterol transport and has antioxidative, anti-inflammatory, antiapoptotic, vasodilative, and antithrombotic activities [43]. Studies performed on transgenic PON1 knockout mice have shown that PON1 prevents atherogenesis [42]. The expression of PON1 is downregulated by reactive oxygen species (ROS) [44]. Oxidized LDL (OX-LDL) triggers macrophages to stimulate anti-inflammatory activity and form foam cells with lipids accumulated in their cytoplasm (Figure 2). Cytokines from adipose tissue contribute to plaque formation in the vascular endothelium (Figure 2). HDL protects LDL against oxidative modification, as observed by less LDL lipid peroxidation in the presence of HDL [42] (Figure 2). PON1, PON2, and PON3 are structurally similar but differ in activity. PON1 is a lactonase and ester hydrolase that degrades thiolactones, xenobiotics, e.g., organophosphate esters, unsaturated aliphatic esters, aromatic carboxylic esters, and carbamates [45]. PON2 and PON3 do not degrade xenobiotics, but have strong lactonase activity [46]. PON2 has an antiatherogenic effect, attributed to the lowering of ROS and apoptosis (Figure 2), and protects mitochondria against oxidative stress [47]. PON3 interacts with apolipoprotein A-1 (apoA1)-HDL similar to that described for PON1, and is strongly associated with microbial diversity and their metabolic pathways, especially the biosynthesis of antioxidant vitamin B1 [46,48]. PON3 is the least studied, and further research is required to determine its exact physiological function [48].

## 5. Gut Microbiota and Inflammation

The gut microbiome of healthy individuals is composed of *Firmicutes*, *Bacteroidetes, Proteobacteria,* and *Actinobacteria.* However, in individuals diagnosed with CVD, *Firmicutes* and *Bacteroidetes* account for approximately 90% of the gut microbiome [49] and they have a higher tendency to produce lipopolysaccharides (LPS) [50]. Hexa-acylated LPS induces inflammation, whereas penta-acylated LPS does not [51]. The binding of microbial LPS and other microbial cell components to Toll-like receptors (TLRs) triggers many inflammatory factors that may lead to CVD. LPS bind to Toll-like receptor 4 (TLR4), bacterial flagella to TLR5, and peptidoglycan to TLR2 [52]. LPS induces inflammation and immune responses that increase intestinal permeability [49]. LPS also promotes the expression of Nod-like receptors (NLRs), especially NLRP3 with its Pyrin domain-containing protein 3 [53]. Upon binding to NLRP3, NLRs activate the nuclear factor kappa-B (NF-κB) or mitogen-activated protein kinase (MAPK) pathways to induce the expression of cytokines and chemokines. This causes a chain reaction inducing an immune inflammatory response [54] leading to CVDs [55,56]. Activation of the TLR4-pathway by LPS leads to the activation of nicotinamide adenine dinucleotide phosphate (NADPH)/ ROS/endothelial nitric oxide synthase (eNOS) and MAPK/NF-κB pathways, leading to endothelial dysfunction and vascular inflammation [57]. A high-fat diet could result in decreased intestinal levels of Gram-positive bifidobacteria and increased LPS-containing gut microbiota, which further leads to obesity, the main risk factor for CVDs [58]. Oxidative stress caused by dysbiosis of gut microbiota promotes the oxidation of LDL to OX-LDL, which inhibits the expression of Endothelial nitric oxide synthase (eNOS), vasoconstriction, and hypertension [59]. Peptidoglycan from bacterial cell walls binds to NOD1 and NOD2, which leads to inflammatory responses and atherosclerosis [56].

Patients with dysbiosis and CVD often develop insulin resistance, lipogenesis, fat accumulation, mitochondrial dysfunction, and systemic or local inflammation [60,61]. These abnormalities lead to altering interactions between gut bacteria and IECs, including the activation of several signaling pathways that regulate host pathophysiological processes such as energy metabolism, local and systemic inflammation, and oxidative stress [62]. Apart from CVD, inflammation may lead to chronic kidney disease (CKD) and angiotensin II-directed hypertension [63,64]. Most research on atherosclerosis and coronary artery disease (CAD) does not differentiate between chronic and acute events. Research on the role gut microbiota plays in acute coronary syndromes needs to be prioritized.

## 6. Short-Chain Fatty Acids and Bile Acids

In many cases, microbial dysbiosis leads to a decrease in short-chain fatty acids (SCFAs) and the disruption of glucose metabolism. Most SCFAs are produced in the colon by *Bifidobacterium, Lactobacillus*, Lachnospiraceae, *Blautia, Coprococcus, Roseburia, Faecalibacterium, Clostridium,* and *Eubacterium* [65,66]. SCFAs adhere to G-protein-coupled receptors (GPCRs), referred to as free fatty acid receptors (FFARs), and regulate histone deacetylase (HDAC) that deacetylates histones and prevents gene expression. G-protein receptor 43 (GPR43/FFAR2) and GPR41 (FFAR3) are located on the surface of IECs [67], neurons of the enteric nervous system (ENS), portal nerve, and sensory ganglia [68,69]. Since GPR41 regulates host energy and promotes glucose and lipid metabolism, a decrease in SCFAs leads to less activated GPR41 and an accumulation of carbohydrates [70].

SCFAs produced in the GIT play an essential role in promoting the secretion of glucagon-like peptide 1 (GLP-1) and peptide YY (PYY) [71]. GLP-1 increases the secretion of insulin [72], and PYY reduces food intake, inhibits intestinal motility, and stimulates bowel movement [70]. Butyrate and propionate, two of the best-studied SCFAs, inhibit tumor necrosis factor (TNF) and NF-κB signaling pathways [73]. Studies conducted on mice have shown that butyrate controls significant components of the tight junction complex in IECs by acting on NLRs [74]. NLRs are essential in the modulation of inflammatory responses [75]. In mice prone to steatohepatitis, butyrate repaired damage to the intestinal mucosa, upregulated zonulin, and lowered endotoxin levels [76].

Butyrate, and to a lesser extent propionate and acetate, engage with GPR41, GPR43, and GPR109a, leading to the activation of MAPK/extracellular signal-regulated kinase (ERK) and p38MAPK signaling pathways, and the generation of extrathymic forkhead box P3 (FOXP3)^+^ Treg cells. This limits inflammation in the GIT and regulates physiological processes in colonocytes [77,78,79]. The engagement of SCFAs with GPR41 promotes the production of interleukin (IL)-22 in CD4^+^ T cells [80]. Butyrate coupled to GPR43 stimulates dendritic cell maturation and induces the activity of retinaldehyde dehydrogenase (RALDH) [81]. The absorption of butyrate to GPR109a enhances retinoic acid production and promotes B cell and IgA responses [82]. SCFAs also signal Th1 cells to activate signal transducer and activator of transcription 3 (STAT3) and mammalian target of rapamycin (mTOR) pathways to produce anti-inflammatory cytokine IL-10 [83]. The role of SCFAs in regulating cytokine production, specifically IL-10 and IL-18, and their role in anti-inflammatory responses are indispensable [78,84]. Additional health-benefitting properties of SCFAs include the control of glucose and cholesterol metabolism [85]. In volunteers with type 2 diabetes, dietary-driven microbial production of SCFAs correlated with clinical improvement in blood glucose control, an increase in GLP-1, and a reduction in acetylated hemoglobin [86].

An increase in mucin-degrading bacteria was noted when gnotobiotic mice were fed a low-fiber diet. This resulted in a reduction in mucus thickness and led to barrier dysfunctions [87,88]. A decrease in SCFA production may lead to serious health issues, including a progressive and irreversible reduction in bacterial diversity over successive generations [89,90]. The diminished ability to degrade fibers from plant material may be rectified by replacing the lost bacterial species. One option is to enrich diets with fermented foods [91]. However, it is important to keep in mind that food–microbe interactions differ between individuals on the same diet and are thus highly personalized [92]. Butyrate acts as a helper in lowering diastolic blood pressure (DPB) [93] and reduces the risk of developing CVD [94].

High-fiber diets consisting of whole grains such as millet, maize, brown rice, sorghum, soya, tapioca, nuts, beans, fibrous vegetables, cashews, watermelon, pineapples, and apples are popular in sub-Saharan countries [95]. These diets support gut microbiota that produce glycan-degrading carbohydrate-active enzymes [91]. The degradation of plant fibers in the colon to SCFAs such as butyrate, propionate, and acetate is important to keep IECs and mucosal layers in a healthy state, ensure proper barrier functions [96], and prevent bacterial translocation [97]. Fructan and galactooligosaccharide (GOS)-rich diets stimulate the growth of *Bifidobacterium* and *Lactobacillus* [98]. Members of these genera are known for the production of SCFAs and their probiotic properties [99,100]. Cereals stimulate the proliferation of *Bacteroides* spp. [101]. According to McDonald et al. [102], the gut microbiome of individuals who consumed more than 30 plant types weekly is dominated by SCFA producers such as *Facealibacterium prausnitzii* and *Oscillospira* spp. The growth of these species is stimulated by acetate-producing *Bifidobacterium* and *Akkermansia* [103]. The composition of a diet determines the type and concentration of SCFAs produced, as shown in a study conducted on ethnic groups in Ghana, Nigeria, Benin, Burkina Faso, Uganda, Kenya, Ethiopia, and South Africa [104]. Findings from this study showed that genera of the order *Lactobacillales* were dominant in fermented cereal and cassava fermentations. Alcoholic beverages contained species of *Lactobacillus*, *Zymomonas,* and *Bacillus*. Diets containing a variety of ingredients support a greater diversity of gut microbiota, especially the alpha group, reviewed by McDonald et al. [102] and Heiman et al. [105].

Bile acids (BAs), produced as a result of microbial metabolism, prevent CVDs by regulating vascular tension and altering ion exchange through cardiomyocyte membranes [106]. Stimulation of the conversion of BAs to free BAs, and secondary BAs by bile salt hydrolase (BSH) and cholesterol 7-alpha hydroxy-lase (CYP7A1) [107], may thus be an alternative way to treat CVDs. Different types of BAs may have opposite effects on Farnesoid receptors (FXRs), which suggests that the regulation of atherosclerosis may rely on much more comprehensive interactions. G protein-coupled receptors (GPCRs) function as cell surface receptors for BAs. Bile acid coupling GPCR1 (GPBAR1), also known as GPCR19, a membrane-type receptor for bile acids (M-BAR) or Takeda G protein-coupled receptor 5 (TGR5), reduces atherosclerotic plaque formation, and thus also plague inflammation, and inhibits phagocytosis of OX-LDL by macrophages [108,109]. Obeticholic acid (OCA or INT-747), a BA-derived agonist, activates FXR and stimulates TGR5 [110]. INT-767 may thus have beneficial therapeutic properties and may reduce atherosclerotic plaques.

Taurocholate alters the mobilization of calcium ions (Ca^2+^) and may lead to irregular contractions of cardiac muscle cells (cardiomyocytes) [32]. Ursodeoxycholic acid (UDCA) prevents re-entrant arrhythmias, causing atrial fibrillation, atrioventricular nodal reentrant tachycardia, atrioventricular reciprocating tachycardia, and ventricular tachycardia [111]. Gut microbiota and their metabolites (especially secondary BAs) play crucial roles in hypertension, atherosclerosis, unstable angina, and heart failure.

## 7. Trimethylamine N-oxide

**Figure 3:**
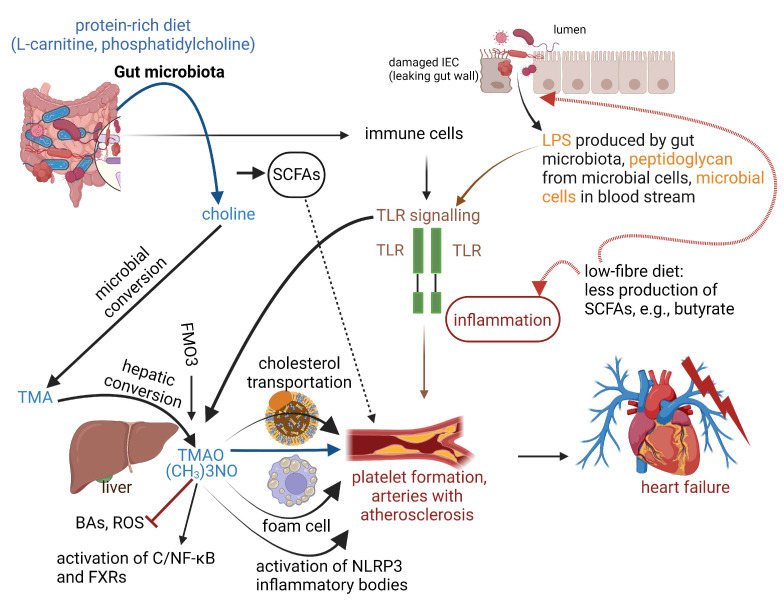
The link between gut microbiota and atherosclerosis. Gut microbiota produces trimethylamine (TMA) from choline, derived from dietary phosphatidylcholine. Choline is microbially converted to TMA and then oxidized to the pro-atherogenic metabolite trimethylamine N-oxide (TMAO). TMAO may contribute to atherosclerosis by interference with cholesterol transportation, foam cell formation, and platelet aggregation. Platelet aggregation leads to atherosclerosis. Disruption of intestinal permeability by damaged intestinal epithelial cells (IECs) results in the leakage of bacterial toxins such as lipopolysaccharides (LPS) formed by microbiota, and cell wall peptidoglycan into the bloodstream. These toxins react with Toll-like receptors (TLRs), leading to systemic inflammation and the aggravation of atherosclerosis. A diet with less dietary fiber decreases the microbial production of short-chain fatty acids (SCFAs) such as butyrate. Butyrate is the main energy source for colonocytes and has an immunomodulatory effect on gut mucosa. Dotted lines denote the effect of diets on IECs and inflammation, lighter circles in the foam cell represent the accumulation of triglycerides, BAs = bile acids, ROS = reactive oxygen species, C/NF-κB = canonical nuclear factor kappa-B, FXRs = Farnesoid receptors, NLRP3 = Nod-like receptor protein 3, FMO3 = Flavin-containing monooxygenase 3. Created using Biorender.com (accessed on 20 August 2024).

Trimethylamine N-oxide (TMAO), produced by gut microbiota from choline, phosphatidylcholine, and l-carnitine, causes vascular endothelial damage by activating nitric oxide dismutase (NOD), leucine-rich repeats (LRR) and pyrin domain-containing NLRP3 inflammatory bodies, increases the release of intracellular calcium ions which activates platelet formation [112], and promotes thrombosis [113] and atherosclerosis, thereby increasing the risk of CVDs [114,115]. TMAO also inhibits the sirtuin 3-superoxide dismutase 2-mitochondrial ROS pathway and the ROS-thioredoxin interactive protein axis [116] and activates the protein kinase C/NF-κB (canonical NF-κB)/vascular cell adhesion molecule-1 pathway, all of which promote atherosclerosis [117] (Figure 3). Furthermore, TMAO inhibits the synthesis of BAs and accelerates the formation of aortic lesions by activating FXRs and small heterodimers [118] (Figure 3). TMAO also upregulates CD36 expression and an ATP-binding cassette transporter A1 in macrophages, resulting in the accumulation of cholesterol [119]. From these findings, TMAO induces systemic inflammation that contributes to the progression of CAD. Exosomes secreted by TMAO-stimulated hepatocytes increase the secretion of TNF-α and IL-6, thus promoting inflammation [120]. TMAO can thus be used as a reporter of atherosclerosis, but also as a target for the prevention and treatment of the disease.

A study that involved 2490 patients with chronic heart failure over almost 10 years showed that elevated levels of TMAO were responsible for heart failure with reduced ejection fraction (HFrEF), a condition ascribed to a 40% or less ejection from the left ventricle, accompanied by progressive left ventricular dilatation and adverse changes in the heart’s size and shape [121]. Flavin-containing monooxygenase 3 (FMO3), produced in the liver, catalyzes the formation of TMAO from gut microbe-derived TMA (Figure 3). The inability to oxidize TMA to TMAO present in humans as trimethylaminuria or “fish odor syndrome”, a rare metabolic disease associated with the accumulation of TMA in a patient’s urine, sweat, and breath. Early studies [122,123,124] have shown that primary trimethylaminuria is linked to mutations in the gene encoding FMO3. It is interesting to note that in cases of potential heart failure, gut microbiota produced more N,N,N trimethyl-5-aminovaleric acid (TMAVA) from the TMAO precursor trimethyl lysine (TML) [125]. Coherent with this, a decrease in the oxidation of fatty acids increases the risk of cardiac hypertrophy and mortality.

Patients suffering from atrial fibrillation (AF) have higher levels of TMAO-producing gut microbiota [126]. The reason why AF patients have higher serum TMAO levels is unknown. We do know that TMAO leads to the instability of the electrophysiology in arteries [127]. TMAO also regulates autonomic nerve conduction through the ganglion plexus and enhances the activation of the NF-κB-mediated inflammatory signaling pathways that induce AF [128] (Figure 3). The precursors of TMAO (choline, betaine, and dimethylglycine) may play an important role. Nevertheless, compelling evidence of changes in gut microbiota and the production of their metabolites points to the development of CVDs [129,130]. LPS can stimulate AF by increasing the activity of the NLRP3 inflammasome (Figure 3). Animal studies [131] have shown that LPS also increases the expression of connexin 43 in cardiomyocytes and the expression of L-type Ca^2+^ channel protein. This decreased myocardium infractions and increased AF. AF may thus be treated by reducing serum levels of LPS. Patients suffering from AF have elevated numbers of *Rumenococcus*, *Streptococcus*, *Enterococcus*, and less *Faecali bacterium*, *Altococcus*, *Oscillobacter*, and *Biliophilus* [132].

## 8. Conclusions

Gut microbiota is involved in CVDs, especially through inflammatory responses, changes in gut barrier integrity, and metabolic processes. CVD, likewise, affects the structure and function of the gut microbiome. In the future, metagenomics and metabolomics will play an important role in studying the relationship between gut microbiota and CVDs. Although the epithelial cellular adhesion molecule (Ep-CAM), trefoil factor 3 (TFF3) peptide, leptin, plasminogen activator inhibitor 1 (PAI1), alpha-1 acid glycoprotein 1 (AGP1), contactin 1 (CNTN1), certain short-chain fatty acids (SCFAs), trimethylamine N-oxide (TMAO), Nod-like receptor NLRP3, elastin, and TMAO are possible biomarkers for CVD, gut microbiota and their metabolites should also be explored. The identification of certain immune responses to CVDs must be studied in-depth. The two-way relationship between gut microbiota and CVD is more complex and involves multiple facets of immune regulation, inflammatory responses, gastrointestinal barrier integrity, and metabolic pathways. Therapeutic interventions concerning dysbiosis of the gut microbiota could be an innovative approach to improve the clinical course of CVDs and heart failure.
